# Abnormal nodal and global network organization in resting state functional MRI from subjects with the 22q11 deletion syndrome

**DOI:** 10.1038/s41598-021-00873-8

**Published:** 2021-11-03

**Authors:** Teuntje A. D. Pelgrim, Matthijs G. Bossong, Analía Cuiza, Luz María Alliende, Carlos Mena, Angeles Tepper, Juan Pablo Ramirez-Mahaluf, Barbara Iruretagoyena, Claudia Ornstein, Rosemarie Fritsch, Juan Pablo Cruz, Cristian Tejos, Gabriela Repetto, Nicolas Crossley

**Affiliations:** 1 Department of Psychiatry, Pontificia Universidad Católica de Chile, Santiago, Chile; 2 Department of Psychiatry, UMC Utrecht Brain Center, Utrecht University, Utrecht, The Netherlands; 3 Departamento de Psiquiatria y Salud Mental, Hospital Clinico Universidad de Chile, Santiago, Chile; 4 Department of Radiology, Pontificia Universidad Católica de Chile, Santiago, Chile; 5 Department of Electrical Engineering, Pontificia Universidad Católica de Chile, Santiago, Chile; 6 Millennium Nucleus for Cardiovascular Magnetic Resonance, Santiago, Chile; 7 Biomedical Imaging Center, Pontificia Universidad Católica de Chile, Santiago, Chile; 8 Genetic and Genomic Center, Universidad del Desarrollo, Santiago, Chile; 9 Escuela de Medicina, Pontificia Universidad Católica, Diagonal Paraguay 362, Santiago, Chile

**Keywords:** Psychosis, Predictive markers, Psychiatric disorders

## Abstract

The 22q11 deletion syndrome is a genetic disorder associated with a high risk of developing psychosis, and is therefore considered a neurodevelopmental model for studying the pathogenesis of schizophrenia. Studies have shown that localized abnormal functional brain connectivity is present in 22q11 deletion syndrome like in schizophrenia. However, it is less clear whether these abnormal cortical interactions lead to global or regional network disorganization as seen in schizophrenia. We analyzed from a graph-theory perspective fMRI data from 40 22q11 deletion syndrome patients and 67 healthy controls, and reconstructed functional networks from 105 brain regions. Between-group differences were examined by evaluating edge-wise strength and graph theoretical metrics of local (weighted degree, nodal efficiency, nodal local efficiency) and global topological properties (modularity, local and global efficiency). Connectivity strength was globally reduced in patients, driven by a large network comprising 147 reduced connections. The 22q11 deletion syndrome network presented with abnormal local topological properties, with decreased local efficiency and reductions in weighted degree particularly in hub nodes. We found evidence for abnormal integration but intact segregation of the 22q11 deletion syndrome network. Results suggest that 22q11 deletion syndrome patients present with similar aberrant local network organization as seen in schizophrenia, and this network configuration might represent a vulnerability factor to psychosis.

## Introduction

The 22q11 deletion syndrome, also known as velo-cardio-facial syndrome, is a genetic multisystem disorder that is caused by a hemizygous microdeletion on the long arm of chromosome 22^1^. It affects approximately 1 in every 1.000–2.000 newborns^2^, being the most frequent chromosomal microdeletion syndrome. Patients may present at a young age with craniofacial, cardiac and immune system deficits, as well as developmental cognitive delays^3–5^. It has also been noted that the deletion confers a higher risk to develop neuropsychiatric disorders later in life, particularly psychotic disorders^6^. About 40% of patients will present a psychotic episode during their lifetime^7^. Studying the brain changes in patients with the 22q11 deletion syndrome could help us understand the high vulnerability to psychosis in this syndrome, as well as inform us about the vulnerability to psychotic disorders such as schizophrenia in general.

Schizophrenia has been described as a dysconnectivity disorder where connections between regions are abnormal, rather than being caused by a localized specific regional disfunction^8–10^. Studies using resting-state functional MRI (fMRI) have shown abnormalities in several connections in patients with schizophrenia, including frontal–temporal connectivity^11,12^, thalamic connectivity^13^ and parietal connectivity^14^, among others. Although only a few studies have investigated functional connectivity in 22q11 deletion syndrome, findings indicate similar abnormal connections as those seen in psychotic patients^15–17^. Network science and graph analysis have also been used to understand the global impact of these connectivity abnormalities found in schizophrenia within an interconnected network such as the brain^18^. The focus of these studies shifts from anatomically localized abnormalities, to changes in the pattern of the whole (brain) network connections. Studies have shown some global properties to be consistently abnormal in schizophrenia, such as a reduction in local cortical organization connectivity and small-worldness^19^, with a preponderance of highly connected regions or hubs to be affected^20^. A few studies have been published using such a network approach on 22q11 deletion syndrome patients, although most of them examined structural networks^21–24^. A previous study using a graph-theory approach focused on the modular organization of the functional network in 22q11 deletion syndrome patients, finding an increased segregation in the network compared to healthy controls, both in children and young adults^25^.

The aim of the present work is to study the whole brain functional network organization of subjects with the 22q11 deletion syndrome and compare them with healthy controls. We first examined the presence of localized anatomical abnormalities, moving then to changes in the network organization at a nodal and global level. We hypothesized that 22q11 deletion syndrome patients would present some abnormalities that are similar to those of patients with schizophrenia. These would involve localized changes in frontal regions and hub regions, as well as a decreased local efficiency, which could be related to their vulnerability to psychosis.

## Methods

### Participants

Participants with the 22q11 deletion syndrome, aged between 15 and 60 years old, were contacted via support groups (“Fundación Chilena del Niño con Síndrome Velocardiofacial'') and from the database of participants from previous studies in our group^26^. Genetic diagnosis of 22q11 deletion syndrome was confirmed using multiplex ligation-dependent probe amplification (MPLA). Subjects with a contraindication for an MRI were excluded. Healthy controls within the same age range, with no lifetime history of a psychotic disorder or other current psychiatric diagnosis were recruited. All healthy controls underwent MLPA to confirm they were not carriers of the deletion. All subjects were assessed using the MINI neuropsychiatric interview^27^ and WAIS-IV^28^. The study was approved by the Ethics committee of the Pontificia Universidad Católica de Chile. Written informed consent was obtained for all subjects or if subjects were under 18, from a parent and/or legal guardian, to participate in accordance with all relevant guidelines and regulations.

### Image acquisition

Structural and functional scans were acquired using a Philips Ingenia 3 T MRI scanner (Philips Healthcare, Best, The Netherlands). For functional images, 200 volumes using a single-shot EPI sequence were acquired while subjects were instructed to remain calm with their eyes opened, with the following parameters: TR = 2.5 s, TE = 32 ms, FA = 82°, FOV = 220 × 220 mm, acquired voxel size = 2.75 × 2.75 × 3.00 mm and number of slices = 40 (continuous descending order). A structural image used in the normalization process in this study was also acquired, namely a 3D TFE T1-weighted sequence was used with an isotropic resolution of 1.0 mm^3^, number of slices = 341, direction of acquisition = sagittal, TR = 7.7 ms, TE = 3.5 ms, FA = 8° and TI = 965.3 ms.

### Image processing

Functional images were preprocessed following previously published pipelines^29^. First, we removed the first four volumes of each acquisition, followed by slice timing correction using SPM12 (Statistical Parametric Mapping, Wellcome Trust Centre for Neuroimaging, UCL, London, UK). All volumes were then realigned following a two-pass realignment, firstly to the first volume and secondly to the mean volume. Functional images were co-registered to the structural image and the nonlinear transform derived from the T1-weighted processing pipeline was applied to co-registered EPI data using ANTs. BOLD timeseries were linearly detrended and EPI data were normalized to mode 1000 units. Then, we applied spatial smoothing with a 6 mm FWHM kernel and bandpass filtering between 0.008 and 0.08 Hz using the fast Fourier transform. We removed the contribution of non-specific (neural) BOLD changes by regressing out the mean white matter and cerebrospinal fluid signals as a denoising procedure^29^. Residual movement was managed using an automated-ICA method^30^.

### Network construction

For each participant, the whole brain was parcellated into 170 regions of interest (ROIs) using an anatomically-based template, namely the third version of the automated anatomical labeling (AAL3) atlas^31^. Time-series were extracted from every ROI, computing a mean time course across all voxels in one region. All cerebellar subregions (26 ROIs) were excluded from our analysis, as our acquisitions did not covered entirely some of these regions. The template also included small regions that in some cases were very noisy due to poor alignment. We therefore excluded ROIs smaller than 57 voxels (228 mm^3^) from the analysis (4 ROIs). 24 small (sub)regions were considered to be important in the pathophysiology of psychosis and 22q11 deletion syndrome, such as the thalamus^16^ and dopaminergic subcortical regions such as the substantia nigra and ventral tegmental areas^32^. We therefore included them after merging subregions within the same anatomical structure (considering as one all subregions of the thalamus, and similarly, combining subregions of the substantial nigra), or merging bilateral regions (ventral tegmental area). Our final network comprised 105 nodes (Supplementary Table 1).

Functional connectivity (FC) matrices were created by computing Pearson correlation coefficients between the mean time course of each ROI and converted to normally distributed *Z*-scores using Fisher's r-to-*Z*-transformation.

### Graph theoretical analysis

To systematically investigate network topology, we included six graph theoretical metrics for each participant. These included three local (nodal) network properties:

- *Weighted degree* measures the total strength of connections that a node has, describing the direct influence of that node and thus reflecting the importance of individual nodes in the network^33^. For node *i*, the weighted degree (*k*) is computed by summing the edge weights (*W*) between *i* and all neighboring nodes:
  1 $${k}_{i}=\sum_{j\epsilon N}{W}_{i, j}$$
- *Nodal efficiency* of node *i (E*_*i*_*)* is computed as the inverse of that node’s average shortest path connecting to all other nodes in the network.
  2 $${E}_{i}= \sum_{i\ne j}\frac{1}{{d}_{ij}}$$
  here d_ij_ is the weighted shortest path between node *i* and *j*.
- *Nodal local efficiency* (*E*_*loc*_) is the average of the inverse of the shortest paths (nodal efficiency *E*) of a subnetwork composed by all the neighboring nodes (*G*_*i*_) of a node *G*:
  3 $${E}_{loc}=\frac{1}{N}\sum_{i\epsilon G}E({G}_{i})$$
  This metric informs us how well the network tolerates faults, by predicting the efficiency of communication among the neighbors of a node when that node has been removed from the network^34^.

We also examined three global metrics:

- *Network local efficiency* is computed as an averaged local efficiency across all nodes in the network and represents the network’s capacity of local information processing.
- *Global efficiency* (*E*_*glob*_) is the inverse of the average shortest path length in the network and is computed as an average of all nodal efficiencies. Global efficiency indicates the effectiveness of information integration across the entire network^34^.
- *Modularity* (*Q*) quantifies the degree to which a network can be subdivided into clearly delineated modules. A highly modular network is divided into non-overlapping subnetworks, maximizing the number of within-modules edges and minimizing between modules edges^35^. We here used a modularity algorithm that maximizes the positive functional connections between regions within a module, but at the same time minimizes the existence of negative functional connections within a module^33^. We here use a weighted version of the algorithm, where the weight of the connection between nodes *i* and *j* is *w*_*ij*_ ∈ {0,1}. Modularity (Q) is therefore defined as:
  4 $$Q =\frac{1}{{v}^{+}}\sum_{i\epsilon N}({w}_{ij}^{+}-{e}_{ij}^{+})\delta {m}_{i}{m}_{j}-\frac{1}{{v}^{+}+{v}^{-}}\sum_{i\epsilon N}({w}_{ij}^{-}-{e}_{ij}^{-})\delta {m}_{i}{m}_{j}$$
  Where the average of *w*_*ij*_ – *e*_*ij*_ examines the difference between existing within-module connections weights (*w*_*ij*_) and within-module connection weights expected by chance (*e*_*ij*_). δ*m*_*i*_*,m*_*j*_ = 1 when *i* and *j* are within the same module or 0 otherwise. The equation examines the positive weights only (+), and on the right side, examines the negative connections (−). Positive and negative connections are weighted differently by their scaling factor, such as that an increase in positive weights would reduce the influence of the negative weights in the modularity partition.

In order to handle anticorrelations of weighted networks^36^, negative edge weights were considered individually to fit each individual graph metric. Absolute values were applied to compute weighted degree, global and local efficiency. Negative edge weights were preserved in matrices to compute modularity as described above. We also performed confirmatory analyses to detect outcome differences when negative edge weights were excluded (transformed to zero).

To allow for comparison of the network organization between groups whose total connectivity strength may differ, we normalized edge weights by the sum of all weights in the network in our computation of global and local efficiency.

Values for all metrics were computed with Matlab custom scripts and Matlab toolbox BCT (Brain Connectivity Toolbox version 2019-03-03, MATLAB)^33^.

### Statistical analyses

Demographic and clinical characteristics were analysed using independent sample *t*‐test for between-group differences in age and IQ and Chi-square test for the categorial variable gender. For all subsequent analyses we applied a general linear model (GLM) analysis including age and gender as covariates of no interest. Since some subjects with the 22q11 deletion syndrome already had a history of psychosis, we included it as another covariate so that results did not reflect the effect of psychosis on network, but rather of the deletion. We also included the mean framewise displacement (FD) as a covariate, which is the mean of the absolute values of the first derivatives of the 6 realignment parameters^37^, and therefore controls our results for potential influence of movement in the scanner. Additional analyses included other factors, specifically mean connectivity value (mean FC) to account for global differences in connectivity strength, as well as IQ. To control for Type I errors of multiple comparisons, false discovery rate (FDR) correction was applied on all *p* values^38^. All statistical analyses were computed using MATLAB (9.8.0.1323502, R2020a) and R (Version 4.0.2^39^).

## Results

### Clinical characteristics of patients

Forty patients with 22q11 deletion syndrome and seventy-six healthy controls were included in the present study (Table 1). No significant differences were detected in age (*p* > 0.05, df = 105, *t*-test) and gender (*p* > 0.05, chi-square) between the patient and control group. All further analyses control for these demographic variables. As expected, patients with the 22q11 deletion syndrome had a lower IQ than healthy controls (*t* (105) =  − 13.52; *p* < 0.001).

**Table 1:** Demographic data of study population.

	Control	22q11 deletion syndrome	Statistics*	*p* value
Participants, *n*	67	40		
Gender, male/female, *n*	43/24	17/23	χ^2^ = 3.759	0.053
Age, years, mean ± SD	23.4 ± 3.7	23.2 ± 8.6	*t* = − 0.169	0.865
History of psychosis, *n*	0	10		
IQ, *n*	55	39		
Mean ± SD	107.3 ± 13.8	69.1 ± 13.0	*t* = − 13.521	< 0.001
**Current antipsychotic medication**				
*n*, DDD				
Aripiprazole	0	4, 100–450		
Olanzapine	0	1, 600		
Quetiapine	0	1, 337.5		

*Between-group differences were tested with a two-sample *t*-test for variables age and IQ and a Chi-square test for gender. Dose equivalents are calculated based on defined daily doses (DDDs).

Nine patients with 22q11 deletion syndrome had a history of psychotic episodes, were receiving antipsychotic treatment, and were considered to be in remission or mildly symptomatic (PANSS mean ± SD = 44 ± 14.2). One extra subject included was acutely psychotic and without medication when assessed (PANSS = 73). As previously described, all subsequent analyses included another covariate to highlight this group.

### Edge-wise differences in network connectivity

**Figure 1:**
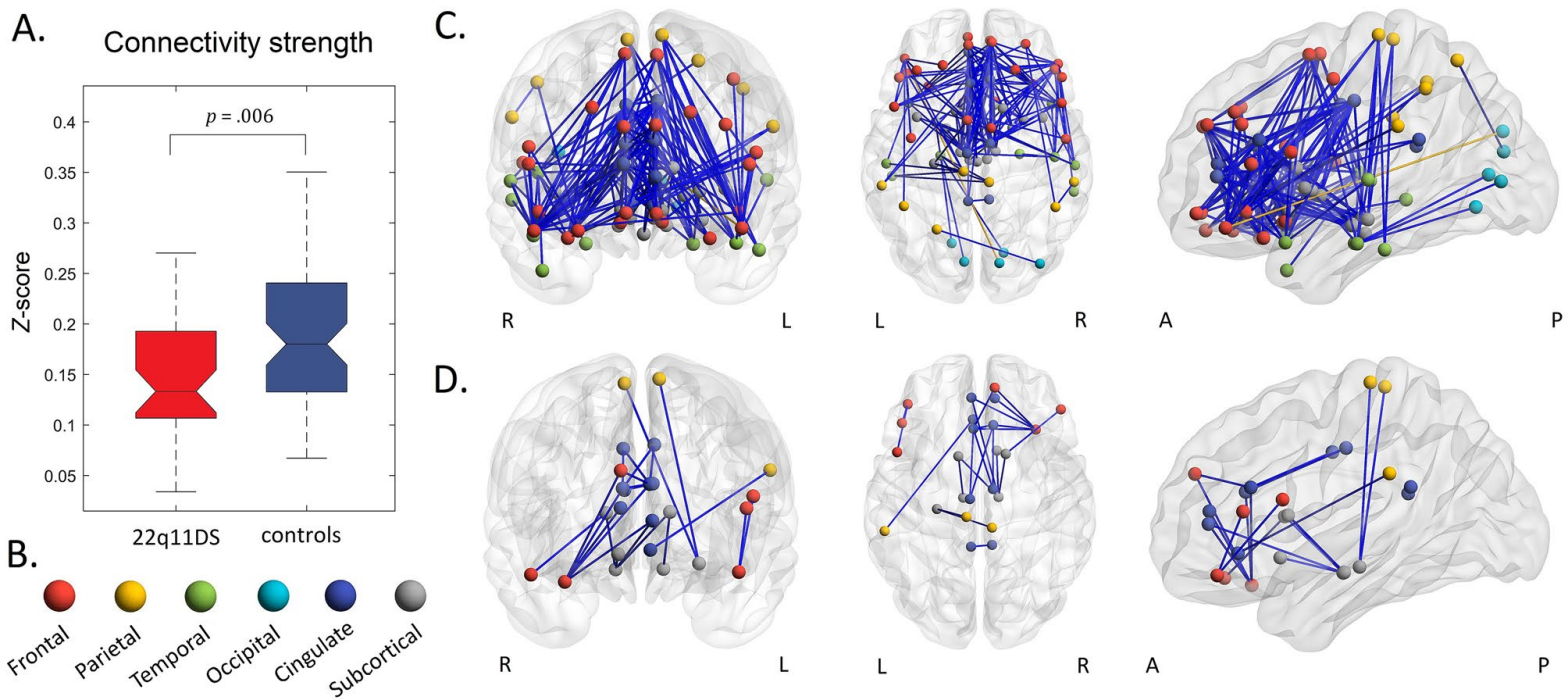
Functional connectivity is reduced in 22q11 deletion syndrome patients. (**A**) Average connectivity strength is lower in patients (*t* (101) = − 2.75, *p* = 0.007). (**B**) Anatomical locations of nodes in the network. (**C**) Functional connectivity (FC) is significantly different in 193 connections, 192 connections (blue) depict lower *Z*-scores in patients, one connection (yellow) is increased in connectivity. Covariates included in the model: age, gender, mean FD and history of psychosis. (**D**) Twenty-three connections are decreased in FC after correcting for an overall lower connectivity in 22q11 deletion syndrome patients. Covariates included the model: age, gender, history of psychosis, mean FD, and mean FC.

There was a significant reduction in the average connectivity strength in 22q11 deletion syndrome patients compared to control subjects (*d* = − 0.53, 95% CI [− 0.92; − 0.15], *t* (101) = − 2.75 *p* = 0.007, Fig. 1A).

Examining strength in individual edges revealed that the observed mean difference in connectivity was driven by a decrease in connectivity strength in a large network composed of 192 connections (P < 0.05 FDR corrected; Fig. 1C). In this network, 21% of reduced connections were cingulate-subcortical, 17% frontal-cingulate, 14% frontal-frontal, 12% frontal–temporal and 8% cingulate-cingulate connections (anatomical locations of all nodes are depicted in Fig. 1B). Remaining edges were mainly distributed within subcortical (6%), between subcortical and frontal (6%) areas, within temporal areas (3%), temporal and cingulate (3%), temporal and parietal (3%) and remaining areas (7%). We further found one connection that was significantly increased in connectivity strength (*p* < 0.05 FDR corrected; Fig. 1C), localized between left lateral orbital gyrus and right cuneus. As seen in Supplementary Fig. 1, this difference is due to a decrease in a connection which in healthy controls is negative.

To address the effect of the overall lower connectivity strength in patients, we included the mean connectivity value (mean FC) as a covariate in the model, revealing a group of 23 reduced connections in 22q11 deletion syndrome (Fig. 1D; Supplementary Table 2). Reductions were mostly localized within cingulate (26%), between frontal and cingulate (22%), within frontal (13%) and within subcortical regions (17%). There were twenty-two connections which were significantly increased after controlling for an overall lower connectivity in patients, mainly composed of inter-hemispheric connections between frontal and occipital brain areas (Supplementary Fig. 2).

### Network properties in 22q11 deletion syndrome

#### Local network properties

**Figure 2:**
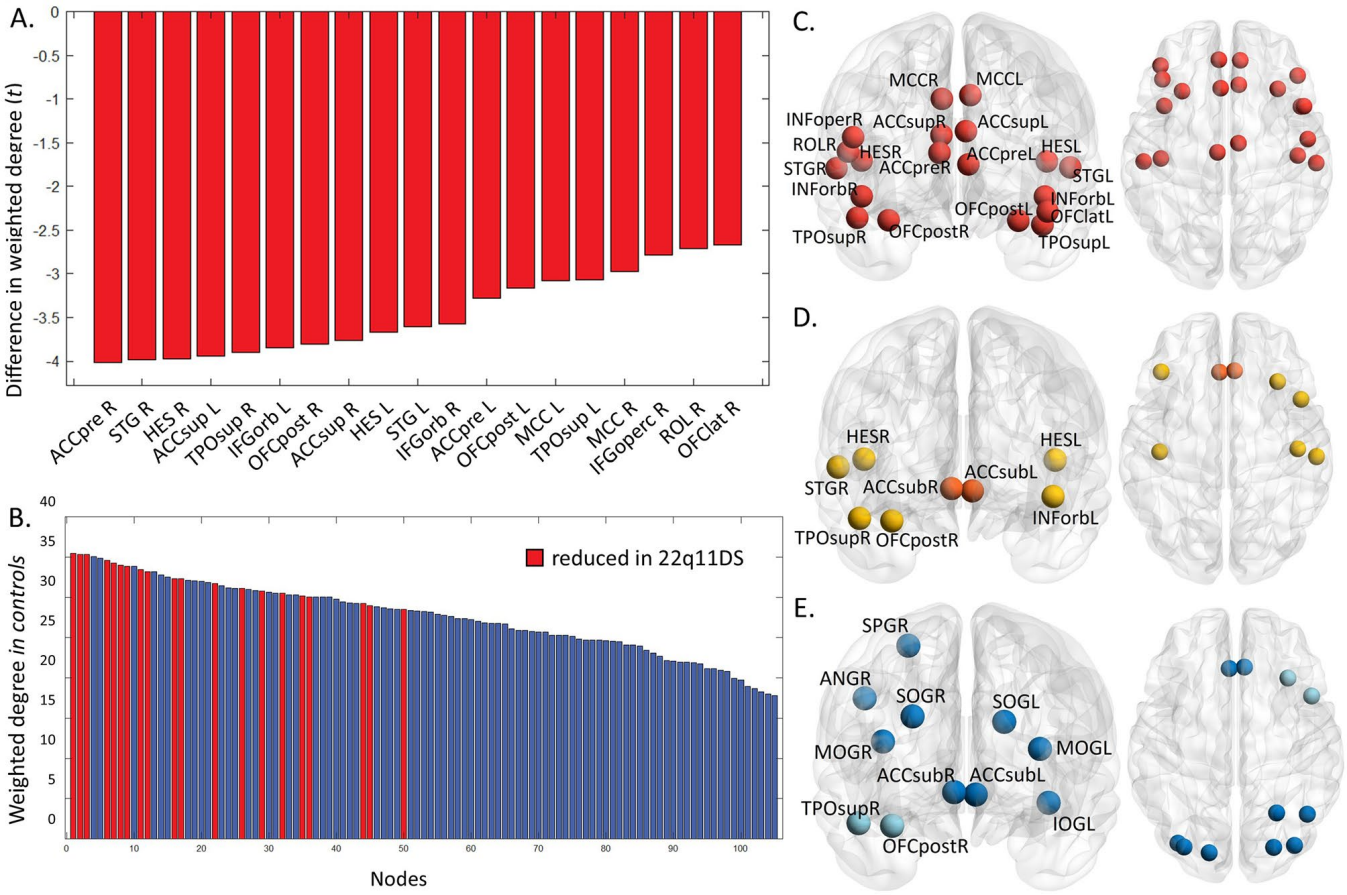
Weighted degree is decreased in highly connected hubs of 22q11 deletion syndrome patients. (**A**) T-values of weighted degree comparison between patients and control subjects, nineteen nodes are significantly reduced after FDR correction. (**B**) Ranking of nodes by average weighted degree in control subjects, nodes with significant lower weighted degree in patients are highlighted in blue. Most affected nodes are depicted on the left side, indicating connector hubs are mostly affected in 22q11DS. (**C**) Physical location of nodal reductions of weighted degree of the 22q11 deletion syndrome network (*p* < 0.05 FDR corrected). (**D**) Abnormal local efficiency in nodes of the 22q11 deletion syndrome network (*p* < 0.05 FDR corrected), reductions are visualized in yellow and increases in orange. (**E**) Nodes in 22q11 deletion syndrome patients presented with abnormal nodal global efficiency, reductions are visualized in light blue and increases in dark blue. WD: weighted degree; eLoc: local efficiency; L: left;: right; TPOsup: temporal pole: superior temporal gyrus; STG: superior temporal gyrus; HES: Heschl’s gyrus; OFCPOST: posterior orbital gyrus; ACCsup: anterior cingulate cortex (supracallosal); ACCpre: anterior cingulate cortex (pregenual); IFGorb: inferior frontal gyrus (opercular part); MCC: middle cingulate; IFGoperc: inferior frontal gyrus, opercular part); ROL: rolandic operculum; SMA: supplementary motor area.

We first examined differences in weighted degree (strength) across groups. After FDR correction, our GLM analysis revealed a significant lower weighted degree in nineteen nodes in the patients’ network compared to controls (Fig. 2A–C). Most affected nodes were localized in frontal (37%), cingulate (31.5%) and temporal areas (31.5%). As shown in Fig. 2B, abnormal nodes were particularly those that were high-degree/high-connectivity hub nodes in healthy subjects.

We further found a significant reduction of local efficiency in six nodes in 22q11 deletion syndrome patients compared to controls, and two nodes that were significantly increased in the patient group (Fig. 2D). The six reduced nodes were also implicated in the weighted degree differences in patients. A lower local efficiency was found in the left inferior frontal gyrus (orbitalis) (*d* = − 0.63, 95% CI [− 1.02; − 0.24], *t* (101) = − 3.26, *p*FDR = 0.027), right posterior orbital gyrus (*d* = − 0.66, 95% CI [− 1.05; − 0.27], *t* (101) = − 3.42, *p*FDR = 0.027), left (*d* = − 0.61, 95% CI [− 0.99; − 0.22], *t* (101) = − 3.13, *p*FDR = 0.030) and right Heschl’s gyrus (*d* = − 0.62, 95% CI [− 1.01; − 0.23], *t* (101) = − 3.21, *p*FDR = 0.027), right superior temporal gyrus (*d* = − 0.63, 95% CI [− 1.02; − 0.24], *t* (101) = − 3.27, *p*FDR = 0.027) and right temporal pole of the superior temporal gyrus (*d* = − 0.77, 95% CI [− 1.16; − 0.38], *t* (101) = − 3.98, *p*FDR = 0.014). Both the left (*d* = 0.62, 95% CI [0.23; 1.01], *t* (101) = 3.21, *p*FDR = 0.027) and right (*d* = 0.68, 95% CI [0.29; 1.07], *t* (101) = 3.50, *p*FDR = 0.027) anterior cingulate cortex (subgenual part) depicted a higher local efficiency in patients.

Our nodal efficiency analysis further revealed a significant increase in nine nodes and a significant reduction in two nodes of the patients’ network after FDR correction. The reduced nodes were localized in frontal and temporal areas, and the increased ones were in occipital, parietal and subcortical areas of the brain (Fig. 2E).

#### Global network properties

We found a significant increase in global efficiency in our 22q11 deletion syndrome group compared to healthy controls (*d* = 0.46, 95% CI [0.08; 0.84], *t* (101) = 2.18; *p* = 0.02). The 22q11 deletion syndrome group global network organization did not differ from that of the healthy control group across segregation graph metrics, as no significant differences were found in modularity (*d* = 0.25, 95% CI [− 0.13; 0.63], t (101) = 1.31; p = 0.19) and network local efficiency (*d* = − 0.29, 95% CI [− 0.67; 0.09], t (101) = − 1.49; p = 0.14). All global metrics are reported in Table 2.

**Table 2:** Group comparisons of global graph metrics in networks of 22q11 deletion syndrome patients and healthy controls

Graph metric	Control (n = 67)	22q11 deletion syndrome (n = 40)	Statistical comparison*	
	Mean ± SD	Mean ± SD	*t* value	*p* value
Network local efficiency	0.00015 ± 0.00003	0.00017 ± 0.00002	− 1.49	0.137
Global efficiency	0.00024 ± 0.00001	0.00025 ± 0.00001	2.38	0.019*
Modularity	0.41469 ± 0.41469	0.51693 ± 0.30664	1.31	0.191

*Covarying for age, gender, history of psychosis and mean FD.

We detected no significant outcome differences of global network properties when negative edge weights were transformed to zero (Supplementary Table 3).

### Covariates of interest

#### IQ

No connections remained significant after FDR correction when including IQ as covariate in all the above-named analyses.

#### History of psychosis

We also explored network changes related to psychosis in the 22q11 deletion syndrome patients. Two connections were increased in connectivity strength in 22q11 deletion syndrome patients who had experienced psychotic symptoms, compared to patients who did not have a history of psychosis and healthy controls at *p* < 0.001. The connections involved were localized between the left middle cingulate and the right anterior cingulate cortex (ACC; subgenual part) (*d* = 0.80, 95% CI [0.41; 1.20], t (101) = 4.18; *p* = 0.0003) and between the right posterior orbital gyrus and the right ACC (pregenual part) (*d* = 0.61, 95% CI [0.22; 0.99], t (101) = 3.14, *p* = 0.0006). However, after FDR correction, results were no longer significant. History of psychosis was not significantly associated with local network properties or global network properties (*p* > 0.05).

## Discussion

The present study was designed to investigate whole-brain functional connectivity in the 22q11 deletion syndrome, particularly looking at local and global topological properties of the functional network using a graph theoretical approach. We found a large network in 22q11 deletion syndrome patients showing a significant decrease in functional connectivity strength, with the most affected connections linking frontal, thalamic, and cingulate regions. We also showed that the 22q11 deletion syndrome network had abnormal local topological properties, as we identified nodal reductions in weighted degree particularly in highly connected hubs and a decreased local efficiency in similar nodes. We found evidence for abnormal whole-brain integration but intact segregation in the 22q11 deletion syndrome network, as global efficiency was increased and modularity and network local efficiency was unchanged.

In line with previous studies of 22q11 deletion syndrome^15–17^, we found alterations in functional connectivity that were widely distributed throughout the brain of patients. On average, whole-brain connectivity strength was significantly lower in 22q11 deletion syndrome patients, which resonates with studies finding globally decreased functional connectivity in schizophrenia^10^. These widespread abnormalities, where most lobes and sub-lobar regions comprised abnormal connections, is in line with the dysconnectivity hypothesis of psychosis^8,9^.

Within this finding of global reduction in connectivity, there were specific regions which were particularly affected. After controlling for an overall lower connectivity strength in patients, we identified significant reductions of connectivity in 25 widely distributed connections, forming a core abnormal network of connections of the 22q11 deletion syndrome brain. These included abnormal connections in frontal regions, particularly in the frontal cingulum, findings which are consistent with previous results in 22q11 deletion syndrome^17,40,41^ and schizophrenia^42–44^. Furthermore, associations between these alterations in frontal connectivity and psychotic symptoms have been described in both groups^45^. We further provide evidence for a disturbed striato-thalamic connectivity in 22q11 deletion syndrome, with lower connectivity of both left and right thalamus with striatal regions (nucleus accumbens and left and right caudate; Supplementary Table 2). Many pathophysiological models of psychosis ascribe a prominent role to the striatum^32^, suggesting that abnormal striato-thalamic connectivity represents cortical disinhibition of subcortical dopamine inducing the emergence of psychotic symptoms^46^. These frontal and striato-thalamic functional connectivity abnormalities, which are similar to those present in schizophrenia but cannot be attributed to a psychotic state (as we controlled for them), could be interpreted as reflecting the genetic vulnerability that predisposes these subjects to psychosis.

Alongside the localized connectivity abnormalities, our results also suggest that the 22q11 deletion syndrome is characterized by aberrant organization in some of the brains’ networks. Specifically, we found nodal reductions in weighted degree (strength), nodal local efficiency and nodal global efficiency, distributed over frontal, cingulate and temporal regions. Nodes which were affected were disproportionately high-degree nodes or hubs in controls. These findings highlight the important role in the network organization that hub nodes have, being involved in many disorders^47^, including schizophrenia^20,48^. A previous study looking at hubs in structural networks in 22q11 deletion syndrome also found abnormalities in these nodes^49^. We also found that many of these hubs had significantly lower local efficiency. Local efficiency could be interpreted as a measure of how much a system is fault tolerant^34^, and therefore the observed decreases in this metric in 22q11 deletion syndrome, particularly in crucial nodes such as hubs, could explain the increased vulnerability to psychosis (allegedly a network failure). Similar efficiency reductions have been found in frontal and temporal brain regions in schizophrenia^50^, and have been associated to psychotic symptom severity in schizophrenia^51,52^. Together, these results provide preliminary evidence of localized brain topology alterations of brain areas that are associated to the vulnerability of psychosis^53^. Our results warrant further study of these nodes, as they could potentially be targeted as biomarkers for outcome prediction for the emergence of psychosis.

In contrast to our hypothesis of a network-wide reduction of local efficiency, only localized reductions were found in patients' brain network. It might well be that whole-brain reduced local efficiency is related to psychosis, but is only present in subnetworks in the vulnerable period, as suggested previously. We did not detect differences in modularity between groups, in contrast to previous findings by Scariati and Schaer^45^. Global efficiency was significantly increased in the patients’ networks, but this finding was not consistent throughout the different analytic approaches as it depended on certain methodological choices related to the negative weights. This result is in contrast to a structural study in which 22q11 deletion syndrome participants presented with longer path lengths (i.e. lower global efficiency)^49^. Examining global efficiency in schizophrenia research has led to contradictory findings, as both, increased^54–56^ and decreased efficiencies^57^ have been found.

The present work has several limitations. We included a relatively low number of subjects (forty). However, despite being the most frequent microdeletion in humans, the 22q11 deletion syndrome is a relatively rare condition with previous studies reporting on a similar -if not fewer- number of patients. Patients with the 22q11 deletion syndrome also present with cognitive impairments including borderline IQ levels. Our results of dysconnectivity and abnormal higher order organization properties might also partly reflect mechanisms of this phenomenon. After including IQ as covariate in our GLM, no result remained significant. Since both, lower IQ and increased vulnerability to psychosis are effects of the deletion, it is not surprising that controlling for one or the other would likely lead to remove much of the brain changes caused by the deletion. We also included a group of 22q11 deletion syndrome participants with a history of psychotic symptoms (*n* = 10). Considering the small sample size, our analyzes were underpowered to detect network differences associated to psychotic symptoms in 22q11 deletion syndrome. Participants with the 22q11 deletion syndrome could have moved more than healthy controls during the image acquisition. Including a measure of in-scanner movement in our analyses such as framewise displacement helped us to account for this.

In conclusion, we here provide further evidence of abnormal connectivity in the 22q11 deletion syndrome, including a global decrease in functional connectivity, particularly in frontal, striatal and cingulate regions. These abnormalities were also accompanied by prominent nodal-level network disorganization, with decreased local efficiency of hub nodes. These network abnormalities could explain the higher vulnerability to psychosis seen in this syndrome.

## Supplementary Information

Supplementary Information.

## Data Availability

The datasets generated and analyzed for the current study are available in the Zenodo repository, https://doi.org/10.5281/zenodo.5234075.
