# Supplementary material for: Abnormal nodal and global network organization in resting state functional MRI from subjects with the 22q11 deletion syndrome
Source: Sci Rep. 2021 Nov 3;11:21623. doi: 10.1038/s41598-021-00873-8 (PMC8566599; doi:10.1038/s41598-021-00873-8)
Supplement: Supplementary file 1 — Supplementary Information. [file 41598_2021_873_MOESM1_ESM.docx]

# Supplemental data for:

**Abnormal nodal and global network organization in resting state functional MRI from subjects with the 22q11 deletion syndrome**

Teuntje A. D. Pelgrim^1,2^, Matthijs G. Bossong^2^, Analía Cuiza^1^, Luz María Alliende^1^, Carlos Mena^1^, Angeles Tepper^1^, Juan Pablo Ramirez-Mahaluf^1^, Barbara Iruretagoyena^1^, Claudia Ornstein^3^, Rosemarie Fritsch^3^, Juan Pablo Cruz^4^, Cristian Tejos^5,6,7^, Gabriela Repetto^8^, Nicolas Crossley^1,6,7*^

^1^Department of Psychiatry, Pontificia Universidad Católica de Chile, Santiago, Chile; ^2^Department of Psychiatry, UMC Utrecht Brain Center, Utrecht University, Utrecht, the Netherlands, ^3^Department of psychiatry, Universidad de Chile, Santiago, Chile; ^4^Department of Radiology, Pontificia Universidad Católica de Chile, Santiago, Chile; ^5^Department of Electrical Engineering, Pontificia Universidad Católica de Chile, Santiago, Chile; ^6^Millennium Nucleus for Cardiovascular Magnetic Resonance, Chile; ^7^Biomedical Imaging Center, Pontificia Universidad Católica de Chile, Santiago, Chile; ^8^Genetic and Genomic Center, Universidad del Desarrollo, Santiago, Chile

**This file includes:**

- **Supplementary Figures:**
  - **Figure S1:** Connection between left lateral orbital gyrus and right cuneus of 22q11DS network significantly increased in functional connectivity
  - **Figure S2:** Relative increases of functional connectivity in the 22q11DS network
- **Supplementary Tables:**
  - **Table S1:** Network construction of anatomical brain regions
  - **Table S2:** Significant connections after covarying for mean FC
  - **Table S3:** Group comparisons of global graph metrics in networks without negative edge weights (transformed to zero) of 22q11DS patients and healthy controls

**Figure S1. Connection between left lateral orbital gyrus and right cuneus of 22q11DS network significantly increased in functional connectivity.** Mean Z-score depicts an increase in functional connectivity in 22q11DS patients between left lateral orbital gyrus and right cuneus. As observed the apparent increase in the connection is due to a decrease in a negative connection present in the healthy brain.


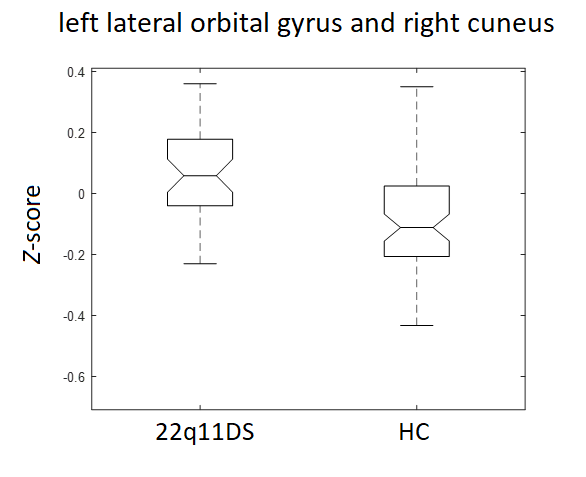


**Figure S2. Relative increases of functional connectivity in the 22q11DS network.** 19 connections of the 22q11DS network are significantly increased in connectivity after correcting for an overall lower connectivity strength in 22q11DS patients.


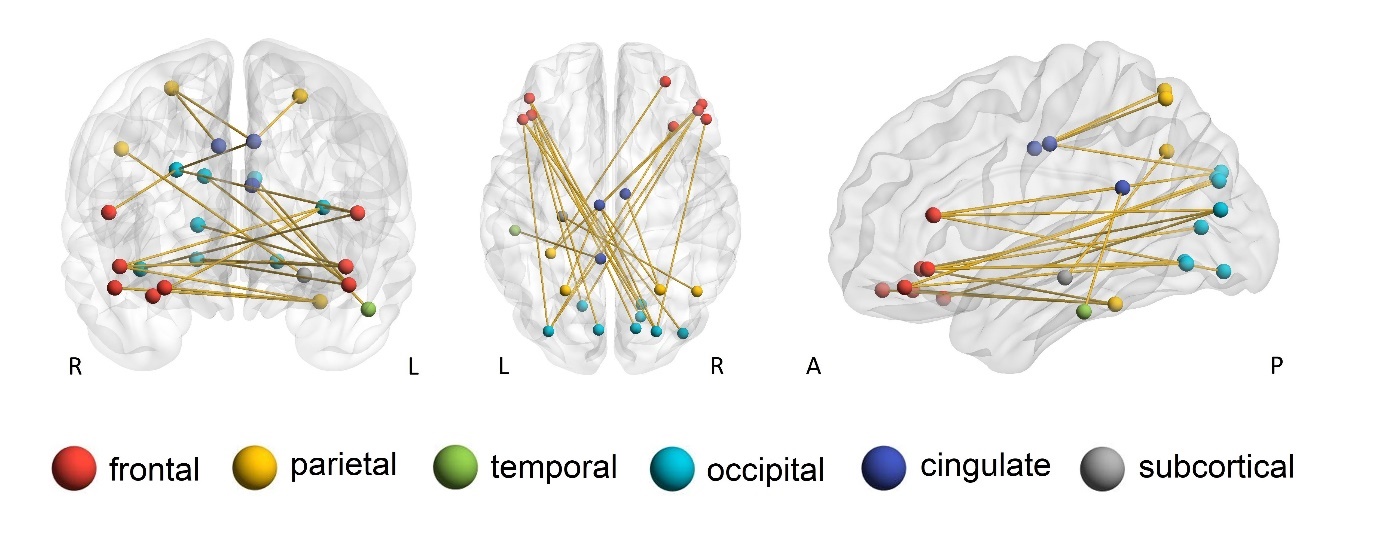


| **Table S1. Network construction of 105 anatomical brain regions.** | | |
| --- | --- | --- |
| **Brain region** | **Comprising sub-regions** | **Lobe or sub lobar structure** |
| PrecentralR | - | Frontal |
| FrontalSup2L | - | Frontal |
| FrontalSup2R | - | Frontal |
| FrontalMid2L | - | Frontal |
| FrontalMid2R | - | Frontal |
| FrontalInfOperL | - | Frontal |
| FrontalInfOperR | - | Frontal |
| FrontalInfTriL | - | Frontal |
| FrontalInfTriR | - | Frontal |
| FrontalInfOrb2L | - | Frontal |
| FrontalInfOrb2R | - | Frontal |
| RolandicOperL | - | Frontal |
| RolandicOperR | - | Frontal |
| SuppMotorAreaL | - | Frontal |
| SuppMotorAreaR | - | Frontal |
| OlfactoryL | - | Frontal |
| OlfactoryR | - | Frontal |
| FrontalSupMedialL | - | Frontal |
| FrontalSupMedialR | - | Frontal |
| FrontalMedOrbL | - | Frontal |
| FrontalMedOrbR | - | Frontal |
| RectusL | - | Frontal |
| RectusR | - | Frontal |
| OFCmedL | - | Frontal |
| OFCmedR | - | Frontal |
| OFCantL | - | Frontal |
| OFCantR | - | Frontal |
| OFCpostL | - | Frontal |
| OFCpostR | - | Frontal |
| OFClatL | - | Frontal |
| OFClatR | - | Frontal |
| InsulaL | - | Subcortical |
| InsulaR | - | Subcortical |
| CingulateMidL | - | Cingulate |
| CingulateMidR | - | Cingulate |
| CingulatePostL | - | Cingulate |
| CingulatePostR | - | Cingulate |
| HippocampusL | - | Subcortical |
| HippocampusR | - | Subcortical |
| ParaHippocampalL | - | Temporal |
| ParaHippocampalR | - | Temporal |
| AmygdalaL | - | Subcortical |
| AmygdalaR | - | Subcortical |
| CalcarineL | - | Occipital |
| CalcarineR | - | Occipital |
| CuneusL | - | Occipital |
| CuneusR | - | Occipital |
| LingualL | - | Occipital |
| LingualR | - | Occipital |
| OccipitalSupL | - | Occipital |
| OccipitalSupR | - | Occipital |
| OccipitalMidL | - | Occipital |
| OccipitalMidR | - | Occipital |
| OccipitalInfL | - | Occipital |
| OccipitalInfR | - | Occipital |
| FusiformL | - | Parietal |
| FusiformR | - | Parietal |
| PostcentralL | - | Parietal |
| PostcentralR | - | Parietal |
| ParietalSupL | - | Parietal |
| ParietalSupR | - | Parietal |
| ParietalInfL | - | Parietal |
| ParietalInfR | - | Parietal |
| SupraMarginalL | - | Parietal |
| SupraMarginalR | - | Parietal |
| AngularL | - | Parietal |
| AngularR | - | Parietal |
| PrecuneusL | - | Parietal |
| PrecuneusR | - | Parietal |
| ParacentralLobuleL | - | Parietal |
| ParacentralLobuleR | - | Parietal |
| CaudateL | - | Subcortical |
| CaudateR | - | Subcortical |
| PutamenL | - | Subcortical |
| PutamenR | - | Subcortical |
| PallidumL | - | Subcortical |
| PallidumR | - | Subcortical |
| HeschlL | - | Temporal |
| HeschlR | - | Temporal |
| TemporalSupL | - | Temporal |
| TemporalSupR | - | Temporal |
| TemporalPoleSupL | - | Temporal |
| TemporalPoleSupR | - | Temporal |
| TemporalMidL | - | Temporal |
| TemporalMidR | - | Temporal |
| TemporalPoleMidL | - | Temporal |
| TemporalPoleMidR | - | Temporal |
| TemporalInfL | - | Temporal |
| TemporalInfR | - | Temporal |
| ACCsubL | - | Cingulate |
| ACCsubR | - | Cingulate |
| ACCpreL | - | Cingulate |
| ACCpreR | - | Cingulate |
| ACCsupL | - | Cingulate |
| ACCsupR | - | Cingulate |
| VentStrL | - | Subcortical |
| VentStrR | - | Subcortical |
| RedNL | - | Subcortical |
| RedNR | - | Subcortical |
| ThalamusL | Left anteroventral nucleus, left lateral posterior, left ventral anterior, left ventral lateral, left ventral posterolateral, left intralaminar, left reuniens, left mediodorsal medial magnocellular, left mediodorsal lateral parvocellular, left lateral geniculate, left medial geniculate, left pulvinar anterior ,left pulvinar medial, left pulvinar lateral, left pulvinar inferior | Subcortical |
| ThalamusR | Right anteroventral nucleus, right lateral posterior, right ventral anterior, right ventral lateral, right ventral posterolateral, right intralaminar, right reuniens, right mediodorsal medial magnocellular, right mediodorsal lateral parvocellular, right lateral geniculate, right medial geniculate, right pulvinar anterior ,right pulvinar medial, right pulvinar lateral, right pulvinar inferior | Subcortical |
| SNL | Left substantia nigra pars compacta, left substantia nigra pars reticulata | Subcortical |
| SNR | Right substantia nigra pars compacta, right substantia nigra pars reticulata | Subcortical |
| VTAbil | Left VTA, right VTA | Subcortical |

| **Table S2. Connections significantly reduced in connectivity strength in 22q11DS.** Significant edge-wise group differences of connectivity strength covarying for mean functional connectivity | | | |
| --- | --- | --- | --- |
| Pairwise brain regions | *t* value * | *p* value | *p*FDR |
| R superior frontal gyrus (medial) ⟷ R posterior orbital gyrus | -4.143 | <.0001 | .023 |
| L inferior frontal gyrus (opercular) ⟷ L lateral orbital gyrus | -4.840 | <.0001 | .005 |
| L inferior frontal gyrus (triangular) ⟷ L lateral orbital gyrus | -4.879 | <.0001 | .005 |
| L posterior cingulate gyrus ⟷ R posterior cingulate gyrus | -4.923 | <.0001 | .005 |
| L hippocampus ⟷ L paracentral lobule | -4.080 | <.0001 | .024 |
| L hippocampus ⟷ R paracentral lobule | -4.11 | <.0001 | .023 |
| R lateral orbital gyrus ⟷ R caudate nucleus | -3.77 | .0003 | .039 |
| L supra-marginal gyrus ⟷ L anterior cingulate cortex (subgenual) | -3.791 | .0002 | .039 |
| R posterior orbital gyrus ⟷ L anterior cingulate cortex (pregenual) | -4.202 | <.0001 | .020 |
| R posterior orbital gyrus ⟷ R anterior cingulate cortex (pregenual) | -4.672 | <.0001 | .008 |
| R superior frontal gyrus (medial) ⟷ L anterior cingulate cortex (sup**) | -4.148 | .0001 | .023 |
| R posterior orbital gyrus ⟷ L anterior cingulate cortex (sup**) | -4.029 | <.0001 | .026 |
| L middle cingulate & paracingulate gyri ⟷ L anterior cingulate cortex (sup**) | -3.997 | .0001 | .026 |
| R middle cingulate & paracingulate gyri ⟷ L anterior cingulate cortex (sup**) | -4.276 | .0001 | .018 |
| R posterior orbital gyrus ⟷ R anterior cingulate cortex (sup**) | -4.211 | <.0001 | .021 |
| L middle cingulate & paracingulate gyri ⟷ R anterior cingulate cortex (sup**) | -4.651 | <.0001 | .008 |
| R middle cingulate & paracingulate gyri ⟷ R anterior cingulate cortex (sup**) | -4.954 | <.0001 | .005 |
| L anterior cingulate cortex (sup**)⟷ R anterior cingulate cortex (sup**) | -3.880 | .0001 | .031 |
| L caudate nucleus ⟷ L thalamus | -4.423 | <.0001 | .013 |
| L caudate nucleus ⟷ R thalamus | -3.768 | .0003 | .039 |
| R caudate nucleus ⟷ R thalamus | -3.909 | .0002 | .031 |
| L anterior cingulate cortex (pregenual) ⟷ R thalamus | -4.086 | <.0001 | .024 |
| R nucleus accumbens ⟷ R thalamus | -3.877 | .0002 | .031 |
| * GLM was fitted to test connectivity strength differences between groups, covarying for age, gender, history of psychosis, mean FD, mean FC. sup** = supracallosal; L = left; R = right; ⟷ = bidirectional connections | | | |

| **Table S3. Group comparisons of global graph metrics in networks without negative edge weights (transformed to zero) of 22q11DS patients and healthy controls** | | | | | | |
| --- | --- | --- | --- | --- | --- | --- |
| Graph metric | Control (n=67) | | 22q11DS (n=40) | | Statistical comparison * | |
|  | Mean ± SD | | Mean ± SD | | *t* value | *p* value |
| Network local efficiency | 0.00019 | ± 0.00002 | 0.00021 | ± 0.00002 | 1.74 | .085 |
| Global efficiency | 0.000275 | ± 0.00003 | 0.00029 | ± 0.00003 | 2.79 | .006* |
| Modularity | 0.246 | ± 0.07 | 0.2707 | ± 0.0682 | 1.42 | .501 |
| *Covarying for age, gender, history of psychosis and mean FD | | | | | | |
